# Comparison of Vascular Function, Cardiometabolic Parameters, Hemorheological Function, and Cardiorespiratory Fitness Between Middle-Aged Korean Women With and Without Obesity—A Pilot Study

**DOI:** 10.3389/fphys.2022.809029

**Published:** 2022-03-29

**Authors:** Hun-Young Park, Won-Sang Jung, Sung-Woo Kim, Kyounghwa Jung, Kiwon Lim

**Affiliations:** ^1^Department of Sports Medicine and Science, Graduate School, Konkuk University, Seoul, South Korea; ^2^Physical Activity and Performance Institute (PAPI), Konkuk University, Seoul, South Korea; ^3^Department of Physical Education, Konkuk University, Seoul, South Korea

**Keywords:** obesity, vascular function, cardiometabolic parameters, hemorheological function, cardiorespiratory fitness

## Abstract

This study aimed to compare vascular function, cardiometabolic parameters, hemorheological function, and cardiorespiratory fitness in middle-aged Korean women according to obesity defined using body mass index (BMI). A total of 32 Korean women aged between 34 and 60 years (16 without obesity, mean age 46.31 ± 7.49 years and 16 with obesity, mean age 49.68 ± 6.69 years) participated in this study. Obesity was defined as BMI ≥ 25 kg/m^2^. The body composition, vascular function, cardiometabolic parameters, hemorheological function, and cardiorespiratory fitness of all participants were measured. Statistical differences in the dependent parameters between individuals with and without obesity were analyzed, and the correlations between BMI and the dependent variables were verified. The obese group showed significantly worse results (*p* < 0.05) for body composition (significantly higher weight, BMI, fat mass, and percent body fat), vascular function [significantly higher branchial ankle pulse wave velocity (baPWV) and lower flow-mediated vasodilation (FMD)], cardiometabolic parameters [significantly higher insulin and homeostatic model assessment for insulin resistance (HOMA-IR)], hemorheological function (significantly lower erythrocyte deformability and higher aggregation), and cardiorespiratory fitness [significantly lower maximal oxygen uptake (VO2max)] compared to the non-obese group. In addition, BMI showed a significant positive correlation (*p* < 0.05) with baPWV (*r* = 0.430); total cholesterol (*r* = 0.376), triglyceride (*r* = 0.411), low-density lipoprotein cholesterol (*r* = 0.462), and insulin (*r* = 0.477) levels; HOMA-IR (*r* = 0.443); and erythrocyte aggregation (*r* = 0.406), and a significant negative correlation (*p* < 0.05) with VO2max (*r* = −0.482) and FMD (*r* = −0.412). Our study confirmed that obesity is a major determinant for deterioration of vascular function, cardiometabolic parameters, hemorheological function, and cardiorespiratory fitness.

## Introduction

Obesity can be classified into primary and secondary obesity according to the cause (Hoelscher et al., 2015). Primary obesity, in which weight and body fat are increased due to an imbalance between energy expenditure and energy intake, is caused by various factors such as diet, lifestyle, age, race, socioeconomic factors, genetics, intestinal microflora, and environmental chemicals (Nammi et al., 2004; Hoelscher et al., 2015). Secondary obesity is caused by genetic disorders, congenital disorders, neuroendocrine disorders, mental disorders, and drugs (Nammi et al., 2004; Hoelscher et al., 2015).

Obesity is recognized as a global health issue and can be described as a new-world syndrome. Obesity is a multi-factorial disorder, which is often associated with many other significant diseases such as hypertension, other cardiovascular diseases, diabetes, osteoarthritis, and certain cancers (Nammi et al., 2004; Park et al., 2018, 2019b). Patients with obesity have a higher risk of morbidity and mortality compared to those with an ideal body weight (Choi, 2012). Many previous studies have shown that a 5–10% reduction in the initial body weight induces significant improvements in a wide range of comorbid conditions in obese patients (Nammi et al., 2004; Wang et al., 2008; Choi, 2012; Jarolimova et al., 2013). In 2016, the World Health Organization reported that more than 1.9 billion adults were overweight and more than 650 million were obese, the prevalence of obesity increased by 50% in 2016 compared to that in 2000, and the prevalence of obesity in women increased by 70% in 2016 compared to that in 2000 (Mohanty and Mohanty, 2021). In South Korea, the prevalence of obesity among adults continued to increase from 29.7% in 2009 to 35.7% in 2018 (Nam et al., 2020).

Previous studies have described in detail the effects of obesity on pathophysiological aspects such as cardiometabolic risk, vascular function, hemorheological function, and cardiorespiratory fitness (Bravo et al., 2006; Vassallo, 2007; Guiraudou et al., 2013; Simonds et al., 2014; Zhang et al., 2014; Schwartz et al., 2017; Cercato and Fonseca, 2019; Bennie et al., 2020). Several cardiometabolic parameters, such as serum glucose, insulin, fatty acid, triglyceride (TG), total cholesterol (TC), low-density lipoprotein cholesterol (LDL-C), and high density lipoprotein cholesterol (HDL-C) levels, and adipocytes are involved in the pathogenesis of obesity, in addition to the gastric and nervous systems that regulate appetite and control food intake (Vassallo, 2007; Zhang et al., 2014; Schwartz et al., 2017; Cercato and Fonseca, 2019). Obesity also induced increased blood pressure and attenuated vascular function. The increase in leptin production due to obesity worsens the function of vascular endothelial cells responsible for vasodilation by interfering with nitric oxide production and activates the sympathetic nervous system to induce vasoconstriction and increase blood pressure (Bravo et al., 2006; Simonds et al., 2014; Cercato and Fonseca, 2019). Guiraudou et al. (2013) verified that obesity is highly correlated with increased red blood cell (RBC) aggregation and blood viscosity due to a rise in hematocrit, and reported that deterioration of hemorheological function increases the risk of diabetes and cardiovascular disease. In addition, they reported that cardiorespiratory fitness was associated with a lower prevalence of obesity, and the associations were more pronounced in groups with higher obesity (Bennie et al., 2020). Another study showed that increased physical activity and improved cardiorespiratory fitness were related to decreased prevalence of obesity and cardiometabolic risk factors (Park et al., 2021b).

However, most previous studies analyzing the relationship between obesity and pathophysiological function were conducted using public data, such as public health surveillance data (Soriano-Maldonado et al., 2016; Park et al., 2020b). Few previous studies have comprehensively reviewed the effects of obesity on dependent variables, such as cardiometabolic risk, vascular function, hemorheological function, and cardiorespiratory fitness, *via* a cross-sectional study based on measured results under strict control.

Therefore, this study aimed to compare vascular function, cardiometabolic parameters, hemorheological function, and cardiorespiratory fitness between middle-aged Korean women with and without obesity. We hypothesized that obesity negatively affects vascular function, cardiometabolic parameters, hemorheological function, and cardiorespiratory fitness, and that body mass index (BMI) is significantly correlated with these dependent variables.

## Materials and Methods

### Participants

Participants were randomly sampled through posters and flyers. A total of 32 Korean women aged between 34 and 60 years (16 without obesity, mean age 46.31 ± 7.49 years and 16 with obesity, mean age 49.68 ± 6.69 years) with no smoking history, orthopedic or cardiovascular diseases, and low levels of activity (<600 MET min/week) in the past year. The menopausal status of the study participants was not assessed. The physical characteristics of the participants are shown in Table 1. Participants were assigned to the non-obese group (*n* = 16; BMI, 22.85 ± 1.05 kg/m^2^) or the obese group (*n* = 16; BMI, 28.28 ± 3.43 kg/m^2^) based on a cutoff value of BMI = 25 kg/m^2^. BMI, a measure of weight corrected for height, reflects the total body fat, and is the most accepted parameter for defining obesity (Taylor et al., 1998). All Korean women completed the study, and their measured data were used in the analyses. We explained the experiments and possible side effects to all participants prior to the start of the study and obtained written consent for participation. The present study was approved by the Institutional Review Board (7001355-201909-HR-334) and was conducted in accordance with the provisions of the Declaration of Helsinki.

**Table 1:** Participants’ characteristics.

Parameters	Non-obese (*n* = 16)	Obese (*n* = 16)	*p*
Age (years)	46.31 ± 7.49	49.68 ± 6.69	0.189
Height (cm)	159.38 ± 5.61	158.96 ± 5.80	0.835
Weight (kg)	58.07 ± 3.91	71.56 ± 9.93	0.000^***^
BMI (kg/m^2^)	22.85 ± 1.05	28.28 ± 3.43	0.000^***^
Free fat mass (kg)	39.39 ± 2.90	43.01 ± 5.63	0.032
Fat mass (kg)	18.68 ± 1.99	28.61 ± 6.68	0.000^***^
Percent body fat (%)	32.15 ± 2.45	39.72 ± 5.07	0.000^***^

Data are means (±SD). SD, standard deviation and BMI, body mass index.

* *p* < 0.05.

** *p* < 0.01.

*** *p* < 0.001 vs. non-obese.

### Study Design

The present study was designed to have 2 consecutive days of testing sessions. On the first testing day, all participants fasted for ≥8 h and after stabilization, the following tests were performed sequentially from 7:00 am: blood pressure (BP) and body composition were measured, and 8 ml of venous blood was collected for the measurement of cardiometabolic parameters and hemorheological function. After sufficient rest and meals, the cardiorespiratory fitness was measured. On the second testing day, pulse wave velocity (PWV) and flow-mediated dilation (FMD), which are indicators of vascular function, were measured in order in the morning (7:00 am) after fasting for ≥8 h (Jung et al., 2020a).

All participants underwent body composition (height, weight, BMI, free fat mass, fat mass, and body fat percentage), vascular function [systolic BP (SBP), diastolic BP (DBP), mean arterial pressure (MAP), pulse pressure (PP), brachial-ankle PWV (baPWV), and FMD], cardiometabolic parameters [TC, HDL-C, LDL-C, TG, free fatty acid (FFA), glucose, and insulin levels; homeostatic model assessment for insulin resistance (HOMA-IR); and homeostasis model assessment of β-cell function (HOMA-β)], hemorheological function (RBC deformability and aggregation), and cardiorespiratory fitness [estimated maximal oxygen uptake (VO_2_max)] measurements.

All measurements were performed in the Konkuk University Metabolic and Environmental Chamber (NCTC-1, Nara control, Seoul, South Korea) at a temperature of 23 ± 1°C and a humidity of 50 ± 5%.

### Body Composition

Body composition parameters including height, weight, BMI, free fat mass, fat mass, and body fat percentage of all the participants were estimated using a stadiometer (YM-1, KDS, Seoul, Korea) and bioelectrical impedance analyzer (Inbody 770, Inbody, Seoul, South Korea). All participants wore lightweight clothing and were asked to remove all metal items from their bodies.

### Vascular Function

Measurement of vascular function was conducted based on previous studies (Jung et al., 2020b; Park et al., 2020b). After the participants rested for at least 20 min, BP (SBP, DBP, MAP, and PP), baPWV, and FMD were sequentially measured to evaluate vascular function.

The BP in the right branchial artery was measured twice using an automatic BP monitor (HBP-9020, Omron, Tokyo, Japan), and the average value was used for analysis. If the results of the first and second measurements were significantly different, the measurement was repeated after a 10-min rest period.

The baPWV was measured using an automatic oscillometer (VP-1000plus, Omron, Osaka, Japan). The VP-1000plus simultaneously records the baPWV and brachial and ankle BPs on the left and right sides, produces an electrocardiogram, and records the heart sounds. Electrocardiography electrodes were placed on both wrists and cuffs were placed on the brachium and ankles bilaterally. A microphone for detecting heart sounds was placed on the left edge of the sternum. The cuffs were connected to a plethysmographic sensor, which determined the volume pulse form and an oscillometric pressure sensor that measured BP. The brachial and ankle pulse volume waveforms were recorded using a semiconductor pressure sensor. The baPWV values on the right and left sides were obtained and averaged for analysis (Park et al., 2020b).

The FMD in the right branchial artery was measured using noninvasive Doppler ultrasound (UNEX-EF, Tokyo, Japan). During FMD measurement, we fixed the ultrasound instrument in the brachial artery region 3–5 cm above the elbow and measured the diameter of the medial muscle artery. Next, blood flow was stopped for 5 min by increasing the cuff pressure by 50 mmHg more than the resting BP. After 5 min, the arterial diameter at deflation was automatically recorded for the next 2 min, and the calculated values of FMD [FMD = (reactive hyperemia diameter − baseline diameter) × 100%] were used to evaluate the diameter and blood flow rate (Jung et al., 2020b).

### Cardiometabolic Parameters

Measurement of cardiometabolic parameters was conducted based on previous studies (Jung et al., 2020a). All cardiometabolic parameters (TC, HDL-C, LDL-C, TG, FFA, glucose, and insulin levels; HOMA-IR; and HOMA-β) were analyzed by the Seegene Medical Foundation (an organization certified by the Korean government). Eight milliliters of venous blood was collected in a serum separating tube (SST). Clot formation was ensured in the SST by centrifuging the sample at 3500 rpm for 10 min. TC level was determined using an enzymatic kinetic assay using Cobas C702 (Roche, Mannheim, Germany). HDL-C and LDL-C levels were detected using a homogeneous enzymatic colorimetric assay using Cobas C702 (Roche, Mannheim, Germany). The glucose level was determined using an enzymatic kinetic assay using Cobas8000 C702 (Roche, Mannheim, Germany), and the insulin level was detected using an electrochemiluminescence immunoassay (ECLIA) using Cobas8000 e602 (Roche, Mannheim, Germany). HOMA-IR and HOMA-β were calculated using the following formula: HOMA-IR = [glucose (mg/dl) × insulin (μU/ml)]/405, HOMA-β = [360 × insulin (μU/ml)/glucose (mg/dl) − 63] (Jung et al., 2020a).

### Hemorheological Function

Measurement of hemorheological function was conducted based on previous studies (Jung et al., 2020a,b; Park et al., 2020b). All participants were evaluated for RBC deformability and aggregation as hemorheological function parameters to evaluate microvascular circulation function. We analyzed RBC deformability and aggregation using Rheoscan-D under environmental conditions of 25°C and 3 Pa shear stress within 4–6 h after blood collection (Uyuklu et al., 2009; Jung et al., 2020a). RBC deformability was measured using the elongation index. For this, we transferred the sample to a 2-mL microseparation tube and diluted it in 700 μl of 5.5% polyvinylpyrrolidone (360 kDa) dissolved in 1 mmol phosphate buffered saline (pH 7.4; osmolality, 300 mOsmol/kg) in a K3-ethylenediaminetetraacetic acid tube (Greiner Bio-one, Chon Nuri, Thailand). Thereafter, 0.5 ml of this solution was analyzed using a D-test kit according to the instructions provided by Rheo Meditech Inc. The accuracy of the RBC elongation index was measured using a Lineweaver–Burk plot model (Baskurt and Meiselman, 2004). RBC aggregation was measured using the aggregation index. For this, we analyzed 8 μl of whole blood using an A-test kit according to the instructions provided by Rheo Meditech Inc. (Kim et al., 2019).

### Cardiorespiratory Fitness

Measurement of cardiorespiratory fitness was conducted based on previous studies (Jung et al., 2020a). All participants were evaluated for estimated VO_2_max as an indicator of cardiorespiratory fitness. A graded exercise test using an electrically braked cycle ergometer (Aerobike 75XLIII, Konami, Japan) was performed to measure the estimated VO_2_max. A heart rate (HR) sensor was attached to the participants’ earlobes and their personal information (height, weight, and age) was entered into the cycle ergometer. Participants performed the graded exercise test after stabilization of their HR. All participants exercised on the cycle ergometer at a rate of 50 rpm until the HR reached 75% of the maximal heart rate (HRmax) using the Lamb protocol (15 W/min; men HRmax: 206–0.69 × age, women HRmax: 205–0.75 × age). The estimated VO_2_max was calculated using the regression equation (estimated VO_2max_ = 9.386 W + 289.6; Miyashita et al., 1985).

### Statistical Analysis

The means and standard deviations were calculated for all dependent parameters. The normality of distribution of all outcome parameters was verified using the Shapiro–Wilk W-test prior to parametric tests. An independent t-test was used to verify the statistical differences between the non-obese and obese groups. Pearson’s correlation analysis was used to analyze the correlations between BMI and all dependent parameters. All analyses were performed using the Statistical Package for the Social Sciences version 23.0 (IBM Corporation, Armonk, NY, United States). *A priori*, the level of significance was set at *p* < 0.05.

## Results

The body composition data between the non-obese and obese groups are presented in Table 1. The obese group showed a significantly higher weight (*p* < 0.001), BMI (*p* < 0.001), fat mass (*p* < 0.001), and body fat percentage (*p* < 0.001) than the non-obese group.

Table 2 presents the vascular function (SBP, DBP, MAP, PP, baPWV, and FMD) data of the non-obese and obese groups. No significant differences between non-obese and obese patients were observed with regard to BP parameters. However, significant differences between both groups were noted in baPWV (*p* = 0.006) and FMD (*p* = 0.024), and the obese group showed a significantly higher baPWV and a lower FMD than the non-obese group.

**Table 2:** Difference in vascular function (mean ± SD) between non-obese and obese.

Parameters	Non-obese (*n* = 16)	Obese (*n* = 16)	*p*
SBP (mmHg)	117.87 ± 11.47	122.71 ± 14.43	0.302
DBP (mmHg)	71.79 ± 6.45	75.25 ± 11.77	0.314
MAP (mmHg)	87.15 ± 7.58	91.07 ± 12.01	0.280
PP (mmHg)	46.07 ± 7.99	47.46 ± 8.85	0.643
baPWV (cm/s)	1169.39 ± 91.79	1318.81 ± 175.34	0.006^**^
FMD (%)	9.81 ± 2.44	7.68 ± 2.62	0.024^*^

SD, standard deviation; SBP, systolic blood pressure; DBP, diastolic blood pressure; MAP, mean arterial pressure; PP, pulse pressure; baPWV, branchial ankle pulse wave velocity; and FMD, flow-mediated vasodilation.

* *p* < 0.05.

** *p* < 0.01.

*** *p* < 0.001 vs. non-obese.

As shown in Table 3, a significant difference between the non-obese and obese groups was found with regard to insulin levels (*p* = 0.015) and HOMA-IR (*p* = 0.022) among cardiometabolic parameters. The obese group showed significantly higher insulin levels and HOMA-IR than the non-obese group. However, there was no significant difference between the groups in the remaining cardiometabolic parameters (TC, HDL-C, LDL-C, TG, FFA, and glucose levels and HOMA-ß).

**Table 3:** Difference in cardiometabolic parameters (mean ± SD) between non-obese and obese.

Parameters	Non-obese (*n* = 16)	Obese (*n* = 16)	*p*
TC (mg/dL)	195.48 ± 46.16	215.75 ± 40.05	0.195
HDL-C (mg/dL)	61.17 ± 11.49	55.39 ± 11.58	0.167
LDL-C (mg/dL)	118.43 ± 44.31	142.99 ± 31.40	0.082
TG (mg/dL)	104.12 ± 32.55	124.77 ± 38.11	0.110
FFA (μEq/L)	874.29 ± 471.43	919.36 ± 340.23	0.759
Glucose (mg/dL)	92.87 ± 12.42	98.98 ± 17.32	0.262
Insulin (mg/dL)	5.46 ± 2.41	7.93 ± 2.98	0.015^*^
HOMA-IR	1.28 ± 0.65	2.00 ± 0.99	0.022^*^
HOMA-β (%)	71.06 ± 25.94	85.47 ± 23.06	0.107

SD, standard deviation; TC, total cholesterol; HDL-C, high-density lipoprotein cholesterol; LDL-C, low density lipoprotein cholesterol; TG, triglyceride; FFA, free fatty acid; HOMA-IR, homeostatic model assessment for insulin resistance; and HOMA-β, *homeostasis model assessment of* β-cell function.

* *p* < 0.05.

** *p* < 0.01.

*** *p* < 0.001 vs. non-obese.

**Figure 1:**
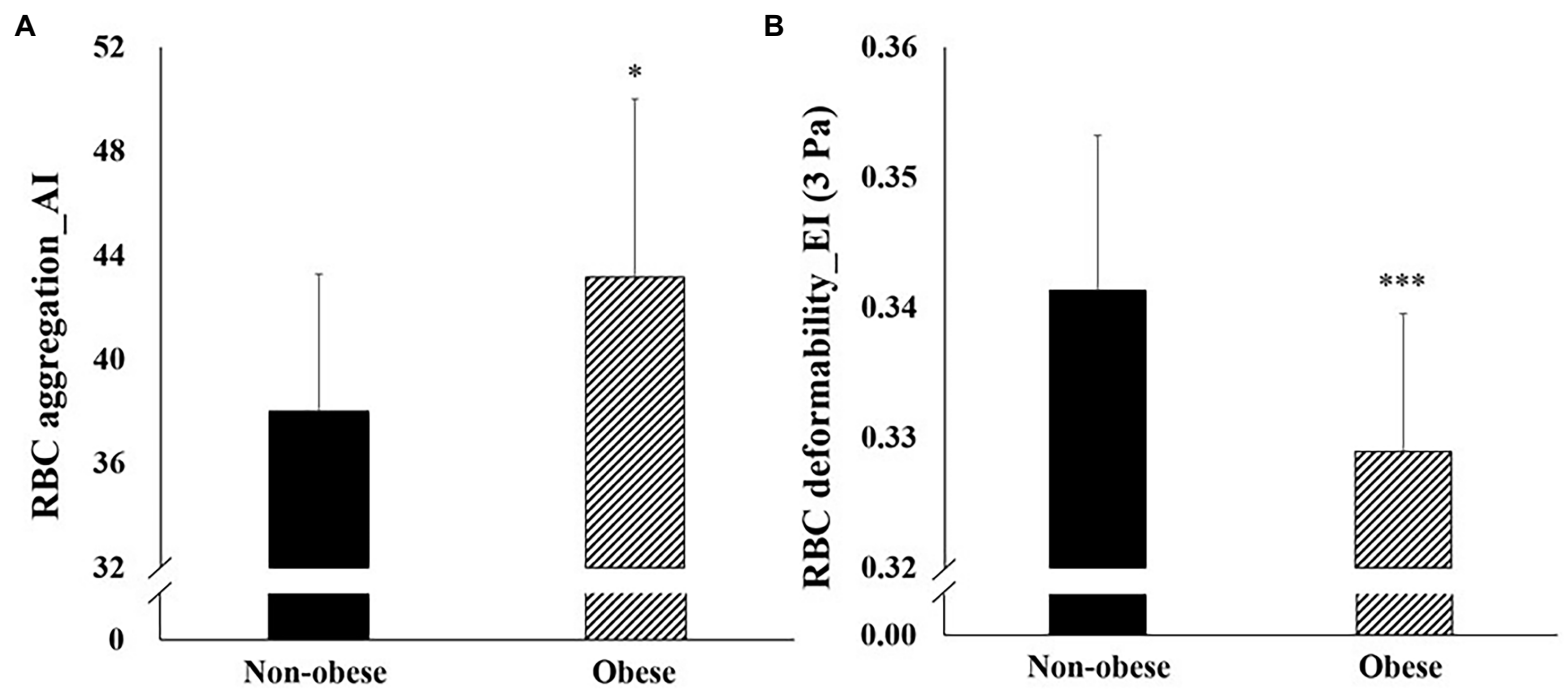
Difference in hemorheological function (mean ± SD) between non-obese and obese. SD, standard deviation; RBC, red blood cell; EI, elongation index; and AI, aggregation index. *p < 0.05; **p < 0.01; and ***p < 0.001 vs. non-obese.

Figure 1 depicts the hemorheological function (RBC deformability and aggregation) data for the non-obese and obese groups. Statistical analysis showed a significant difference in RBC deformability (*p* = 0.024) and aggregation (*p* = 0.004) between the groups. The obese group showed significantly lower RBC deformability and higher RBC aggregation than the non-obese group.

**Figure 2:**
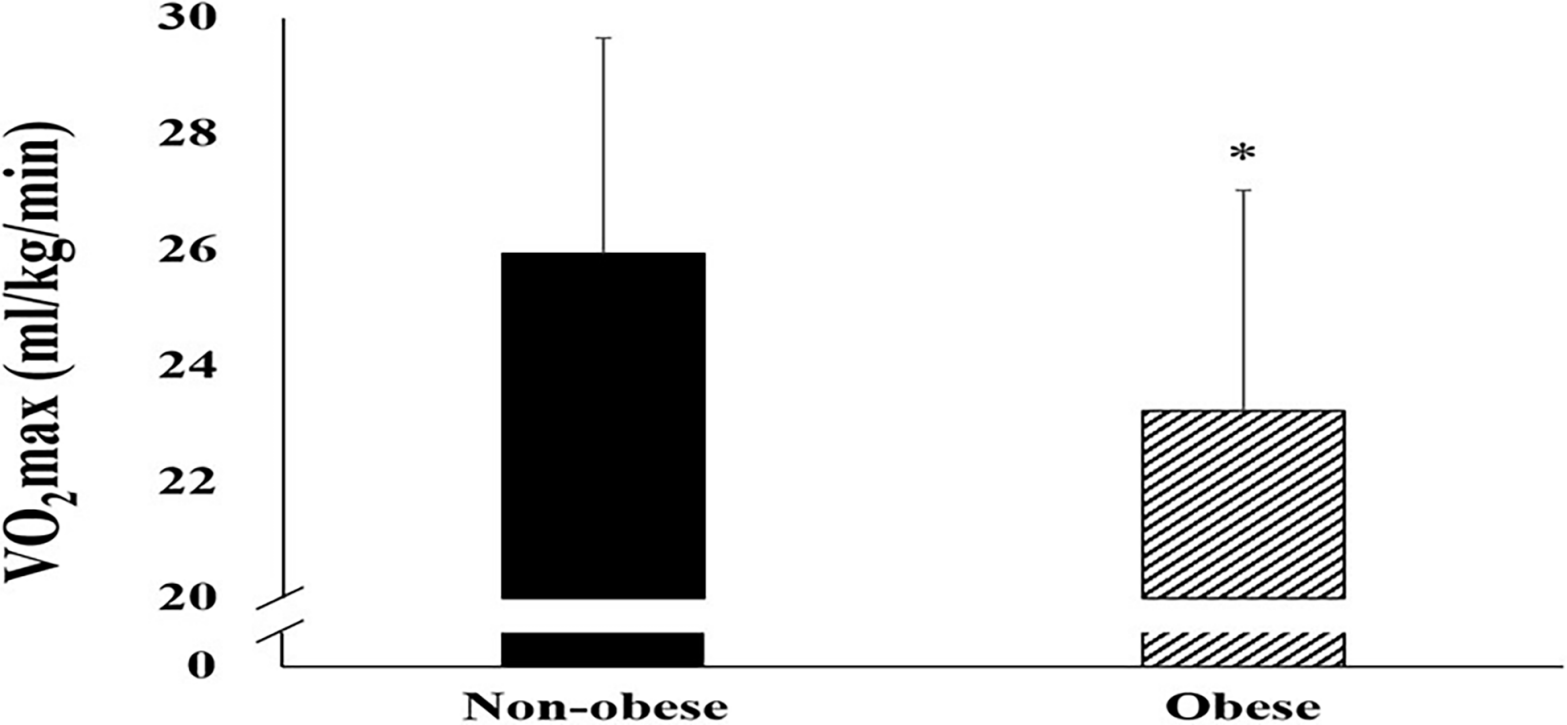
Difference in maximal oxygen uptake (mean ± SD) between non-obese and obese. SD, standard deviation and VO_2_max, maximal oxygen uptake. *p < 0.05; **p < 0.01; ***p < 0.001 vs. non-obese.

The cardiorespiratory fitness in both groups is shown in Figure 2. A significant difference between the non-obese and obese groups was observed in VO2max (*p* = 0.049). The obese group showed significantly lower VO2max than the non-obese group.

**Figure 3:**
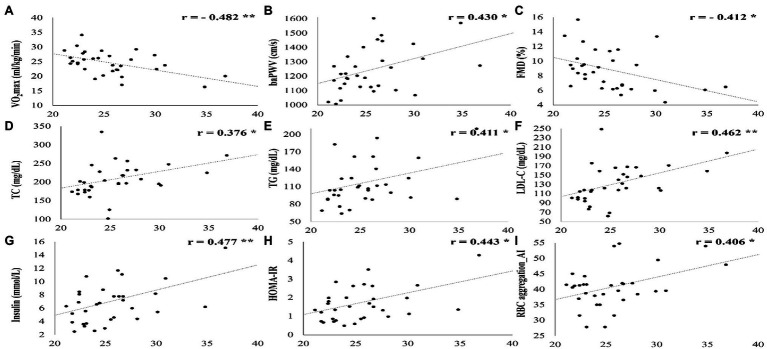
All parameters that showed a significant correlation with BMI. SD, standard deviation; VO_2_max, maximal oxygen uptake; baPWV, branchial ankle pulse wave velocity; FMD, flow-mediated vasodilation; TC, total cholesterol; LDL-C, low density lipoprotein cholesterol; HOMA-IR, homeostatic model assessment for insulin resistance, RBC, red blood cell; and AI, aggregation index. *p < 0.05; **p < 0.01; ***p < 0.001.

Figure 3 shows a significant correlation between the vascular function, cardiometabolic parameters, hemorheological function, and cardiorespiratory fitness and BMI. BMI was significantly negatively correlated with VO_2_max (*r* = −0.482, *p* = 0.005) and FMD (*r* = −0.412, *p* = 0.019). On the other hand, BMI was significantly positively correlated with baPWV (*r* = 0.430, *p* = 0.014), TC (*r* = 0.376, *p* = 0.034), TG (*r* = 0.411, *p* = 0.020), LDL-C (*r* = 0.462, *p* = 0.008), insulin (*r* = 0.477, *p* = 0.006), HOMA-IR (*r* = 0.443, *p* = 0.011), and RBC aggregation (*r* = 0.406, *p* = 0.021).

## Discussion

In the present study, we hypothesized that individuals with obesity may show attenuated vascular function, cardiometabolic parameters, hemorheological function, and cardiorespiratory fitness compared with individuals without obesity and that BMI may be significantly correlated with these dependent variables. Our study verified that obesity is a major risk factor for the deterioration of vascular function (a higher baPWV and a lower FMD), cardiometabolic parameters (higher insulin levels and HOMA-IR), hemorheological function (lower RBC deformability and higher aggregation), and cardiorespiratory fitness (lower VO_2_max). We also demonstrated that BMI was significantly correlated with various pathophysiological parameters (negatively correlation: VO_2_max and FMD; positively correlation: baPWV, TC, TG, LDL-C, insulin, HOMA-IR, and RBC aggregation).

Regarding the relationship between BMI and body composition, Ranasinghe et al. (2013) analyzed the relationship between BMI and body fat percentage in a group of South Asian adults who had a different body composition than the ethnic group in the present study. They reported that BMI shows a strong correlation with the body fat percentage measured using bioelectrical impedance in this sub-population of South Asian adults. On the other hand, Meeuwsen et al. (2010) examined the relationship between age, sex, and the influence of age and sex on BMI and body fat percentage over a wide range of BMI and age, and reported that the association between BMI and body fat percentage is not strong. Particularly, in the desirable BMI range, it is curvilinear rather than linear and is affected by age. Although our study was a pilot study with a small sample size, we confirmed a significant difference in the fat mass and body fat percentage between the non-obese and obese groups, and a strong correlation between BMI and fat mass or body fat percentage. These results are consistent with those of a previous study (Ranasinghe et al., 2013).

Obesity promotes the development of several major cardiovascular risk factors (Park et al., 2019a). Obesity may impair vascular endothelial function, a key factor in the pathogenesis of atherosclerosis and in triggering acute ischemic events, and is associated with increased BP and attenuated vascular function (Brook, 2006). Especially, increased leptin secretion from adipocytes in obese individuals attenuates vascular endothelial function, which is responsible for vasodilation, by interfering with nitric oxide production and activates the sympathetic nervous system to induce vasoconstriction and increase BP (Bravo et al., 2006; Brook, 2006; Simonds et al., 2014; Cercato and Fonseca, 2019). Therefore, we investigated the difference in BP, baPWV (a predictive indicator of arterial stiffness), and FMD (vasodilation of arteriole due to the release of nitric oxide from endothelial cells that occurs when blood flow in it increases) between the non-obese and obese groups. Tang et al. assessed the association between BMI and baPWV in Chinese individuals who were overweight/obese and reported that individuals who were overweight/obese had significantly higher baPWV than those with normal weight (Tang et al., 2020). Son et al. (2017) analyzed the effect of obesity on BP and PWV in middle-aged Korean women and reported that obesity is closely related to blood pressure and arterial stiffness. Davison et al. (2010) investigated the relationship between obesity and arterial endothelial function and found that the obese group showed a lower FMD than the non-obese group, and FMD was impaired in patients with obesity, which may contribute to arterial and metabolic dysfunction. Ne et al. (2017) analyzed the relationship between obesity and arterial endothelial function using a meta-analysis, and demonstrated that obesity was associated with lower flow-mediated dilatation. In the present study, the obese group showed a higher PWV and lower FMD than the non-obese group, and BMI was significantly correlated with PWV and FMD, which is consistent with the results of previous studies. These results suggest that obesity induces arterial stiffness and deteriorates endothelial function.

Many previous studies have clinically confirmed the relationship between obesity and cardiometabolic parameters. Soriano-Maldonado et al. (2016) analyzed the effect of severe/morbid obesity (BMI ≥ 35) on cardiometabolic parameters and concluded that severe/morbid obesity is highly correlated with more adverse cardiometabolic risk factors in both male and female adults. Nyangasa et al. (2019) investigated the association between obesity indices and cardiovascular risk factors and reported that the obese group showed higher BP, LDL-C levels, and glycated hemoglobin levels and lower HDL-C levels than the non-obese group. They concluded that increased BMI, waist circumference, and body fat percentage were strongly associated with hypertension, with individuals with high waist circumference being twice as likely to have hypertension. In our study, only insulin levels and HOMA-IR among the cardiometabolic parameters were higher in the obese group than in the non-obese group. This can be explained by the fact that the BMI of the obese group in the present study was 28.28 ± 3.43, and the participants did not have severe obesity. However, as there was a significant correlation between BMI and most cardiometabolic parameters (TC, TG, LDL-C, and insulin levels and HOMA-IR), it was confirmed that an increase in BMI could increase metabolic disease, as reported in previous studies.

RBC deformability is a key feature of erythrocytes that allows them to pass through blood vessels smaller than themselves, and decreased RBC deformability results in decreased blood flow to the capillaries, which can result in a variety of microvascular diseases (Shin et al., 2009; Kim et al., 2019). In contrast, erythrocytes tend to aggregate to form linear stacking shapes known as rouleaux and exacerbated RBC aggregation in the microcirculation by excessive rouleaux formation causes diabetic microcirculatory diseases (Kim et al., 2019; Lee et al., 2019). Wiewióra et al. (2010) compared hemorheological properties between non-obese and obese groups, and examined the correlation between BMI and hemorheological parameters to determine the relationship between obesity and hemorheological disturbances. They reported that obesity may attenuate hemorheological properties by increasing whole blood viscosity and RBC aggregation. We investigated the effect of obesity, which was defined as BMI ≥ 25, on hemorheological properties and the correlation between BMI and hemorheological function. Our results showed that obesity was significantly correlated with RBC deformability and aggregation, and obesity induced deterioration of hemorheological function *via* decreased RBC deformability and increased RBC aggregation. These results are consistent with those of previous studies that reported that changes in rheological properties of blood are closely related to obesity and diabetic microcirculatory diseases (Shin et al., 2009; Wiewióra et al., 2010; Kim et al., 2019; Lee et al., 2019).

Cardiorespiratory fitness reflects the ability of the cardiorespiratory system to transport oxygen to skeletal muscles during exercise and is the most important clinical indicator for evaluating pathological conditions, including metabolic and cardiovascular diseases (Ross et al., 2016; Park et al., 2021a). Cardiorespiratory fitness is an index that reflects risk factors and disease burden over an individual’s lifetime, and a low cardiorespiratory fitness increases the risk of obesity, diabetes, hypertension, and cardiovascular disease, thereby increasing morbidity and mortality (Kodama et al., 2009; Gray et al., 2015; Park et al., 2021a). Therefore, we evaluated the correlation between BMI and VO_2_max, and analyzed the difference in VO_2_max between the non-obese and obese groups. We observed that the obese group showed a lower VO_2_max than the non-obese group, and BMI had a significant negative correlation with VO_2_max. These results are consistent with those of previous studies that showed that obesity worsens cardiorespiratory fitness, which increases the prevalence of diabetes, hypertension, and cardiovascular disease, thereby increasing morbidity and mortality (Kodama et al., 2009; Gray et al., 2015; Park et al., 2020a, 2021a).

## Limitations

Our study confirmed the normal distribution using the Shapiro–Wilk W-test, its limitation was the relatively small sample size. Thus, to utilize the results of this study in the field of public health, it is necessary to comprehensively evaluate the correlation between vascular function, cardiometabolic parameters, hemorheological function, and cardiorespiratory fitness and obesity (BMI ≥25) using a larger sample size. Also, we included only adult women in the study. Men and women have different physiological characteristics and need further research on adult men. Our study also did not evaluate extraneous variables that could affect dependent variables such as the ovarian cycle, female sex hormones, diet, and exercise.

## Conclusion

In conclusion, the present study suggests that obesity, defined as BMI ≥25 kg/m^2^, is a major risk factor that deteriorates vascular function, cardiometabolic parameters, hemorheological function, and cardiorespiratory fitness, and BMI is significantly correlated with these dependent variables in adult women.

## Data Availability Statement

The original contributions presented in the study are included in the article/supplementary material, further inquiries can be directed to the corresponding author.

## Ethics Statement

The studies involving human participants were reviewed and approved by the Institutional Review Board (7001355-201909-HR-334) and was conducted in accordance with the provisions of the Declaration of Helsinki. The patients/participants provided their written informed consent to participate in this study.

## Author Contributions

H-YP: conception and study design, investigation, and writing-original draft preparation. S-WK, W-SJ, and KJ: statistical analysis, data interpretation, and writing-review and editing. KL: supervision. All authors contributed to the article and approved the submitted version.

## Funding

This work was supported by the Ministry of Education of the Republic of Korea and the National Research Foundation of Korea (NRF-2019S1A5B8099542).

## Conflict of Interest

The authors declare that the research was conducted in the absence of any commercial or financial relationships that could be construed as a potential conflict of interest.

## Publisher’s Note

All claims expressed in this article are solely those of the authors and do not necessarily represent those of their affiliated organizations, or those of the publisher, the editors and the reviewers. Any product that may be evaluated in this article, or claim that may be made by its manufacturer, is not guaranteed or endorsed by the publisher.
